# *Pittosporum qibainongense* (Pittosporaceae), a new species from karst areas of southwest China

**DOI:** 10.3897/phytokeys.271.185044

**Published:** 2026-03-12

**Authors:** Yu-Song Huang, Zhao-Cen Lu, Yan Liu, Yun-Fei Deng

**Affiliations:** 1 Guangxi Key Laboratory of Functional Phytochemicals Research and Utilization, Guangxi Institute of Botany, Guangxi Zhuang Autonomous Region and the Chinese Academy of Sciences, Guilin, 541006, Guangxi, China Guangxi Key Laboratory of Functional Phytochemicals Research and Utilization, Guangxi Institute of Botany, Guangxi Zhuang Autonomous Region and the Chinese Academy of Sciences Guilin China; 2 State Key Laboratory of Plant Diversity and Specialty Crops, South China Botanical Garden, Chinese Academy of Sciences, Guangzhou, 510650, Guangdong, China Guangxi Key Laboratory of Plant Conservation and Restoration Ecology in Karst Terrain, Guangxi Institute of Botany, Guangxi Zhuang Autonomous Region and Chinese Academy of Sciences Guilin China; 3 University of Chinese Academy of Sciences, Beijing, 100049, China South China Botanical Garden, Chinese Academy of Sciences Guangzhou China; 4 Guangxi Key Laboratory of Plant Conservation and Restoration Ecology in Karst Terrain, Guangxi Institute of Botany, Guangxi Zhuang Autonomous Region and Chinese Academy of Sciences, Guilin, 541006, Guangxi, China University of Chinese Academy of Sciences Beijing China; 5 Key Laboratory of National Forestry and Grassland Administration on Plant Conservation and Utilization in Southern China, Guangzhou, 510650, Guangdong, China Key Laboratory of National Forestry and Grassland Administration on Plant Conservation and Utilization in Southern China Guangzhou China

**Keywords:** Endangered species, flora of Guangxi, morphology, *
Pittosporum
*, taxonomy

## Abstract

*Pittosporum qibainongense* Y.S.Huang, Yan Liu & Y.F.Deng, a new species of Pittosporaceae, is described and illustrated from karst areas of southwest China. It is morphologically similar to *P. trigonocarpum* in having leaves clustered at the branchlet apex, blades obovate or oblong-lanceolate, terminal umbellate inflorescences, and capsules dehiscing by three valves, but it can be easily distinguished from the latter by its leaf margins strongly revolute when dry, lateral veins conspicuous on both surfaces, placentas at the lower middle part, and capsules usually multiple, ovate or obovoid, and glabrous. Photographs of this new species, as well as a table and a key to distinguish it from the similar species, are provided. The threatened status of the new species is also assessed.

## Introduction

*Pittosporum* Banks ex Gaertner (1788: 286) is a genus in the family Pittosporaceae and comprises 255 species widely distributed in the tropical and subtropical Old World and the Pacific (Zhang et al. 2003; Carolin and Bittrich 2018; POWO 2026). Asia is an important center of diversity of *Pittosporum*, and China has the most abundant species among Asian countries (Gowda 1951; Zhang et al. 2003; Huang et al. 2024). The most recent revision of the genus was carried out in the “Flora of China”, and 48 species with 18 varieties of *Pittosporum* were recognized. In China, the genus is mainly distributed in Yunnan, Guangxi, Guizhou, and Sichuan provinces, among which 35 species and 15 varieties are endemic (Zhang et al. 2003). Afterward, two species were described (Chen et al. 2021; Huang et al. 2024).

*Pittosporum* is a confusing and difficult genus in taxonomy, with most species morphologically variable and lacking distinguishing features for quick identification (Gowda 1951). During a field survey in Dahua County, Guangxi Zhuangzu Zizhiqu, China, in November 2023, a fruiting plant was found. It is similar to *P. trigonocarpum* H. Léveillé (1913: 492) in leaf morphology and habitat, but it can be easily distinguished from the latter by its leaf margins strongly revolute when dry, capsules ovate or obovoid and glabrous, and funicle at the lower middle part. For further identification, in April 2024, a field survey was conducted, and flowering material was collected. After consulting the relevant literature (Gowda 1951; Chang and Yan 1974, 1978, 1979; Peng and Deng 2001; Zhang et al. 2003), as well as conducting detailed comparisons with other similar species of this genus, it is confirmed that it represents a new species and is described and illustrated below.

## Materials and methods

Field surveys were conducted during the flowering and fruiting seasons at the type locality and its vicinity. The morphological data were obtained based on specimens collected from the field. Voucher specimens are deposited in the Herbaria of Guangxi Institute of Botany (**IBK**), South China Botanical Garden, Chinese Academy of Sciences (**IBSC**), and Institute of Botany, Chinese Academy of Sciences (**PE**).

Comparisons between this new species and its similar species were based on descriptions from protologues and examination of herbarium specimens or images of specimens (including types) at IBK, GXMG, GXMI, IBSC, SYS, PE, KUN, GFS, GZAC, and TIE. The assessment of the threatened status of this new species is based on the IUCN Red List of Threatened Species Categories and Criteria and Guidelines for Using the IUCN Red List Categories and Criteria (IUCN 2012, 2024).

## Taxonomic treatment

### Pittosporum qibainongense

Taxon classificationPlantaeApialesPittosporaceae

Y.S.Huang, Yan Liu & Y.F.Deng sp. nov.

94674BEB-32B0-5447-97A9-D13C581C11F9

urn:lsid:ipni.org:names:77377651-1

Figs 1, 2, 3 (A–F)

#### Diagnosis.

The new species is morphologically similar to *P. trigonocarpum* H. Lév. in leaves clustered at branchlet apex, blades obovate or oblong-lanceolate, umbellate inflorescences terminal and capsules usually dehiscing by 3 valves, but can be easily distinguished from the latter by lateral veins 6–9 pairs, conspicuous on both surfaces (vs. 6 pairs, inconspicuous on both surfaces), petioles 6–8 mm long (vs. ca. 1 cm long), pedicels 1–1.5 cm long, glabrous or sparsely puberulous (vs. 1–2.5 cm long, glabrous), capsules multiple, rare solitary, ovate or obovoid, glabrous (vs. solitary, triangular or globose, puberulous), seeds 10–25, 6–8 mm long (vs. 9–15, 5–6 mm long), and funicle at lower middle part of placenta (vs. scattered on the elongated placenta).

**Figure 1.**
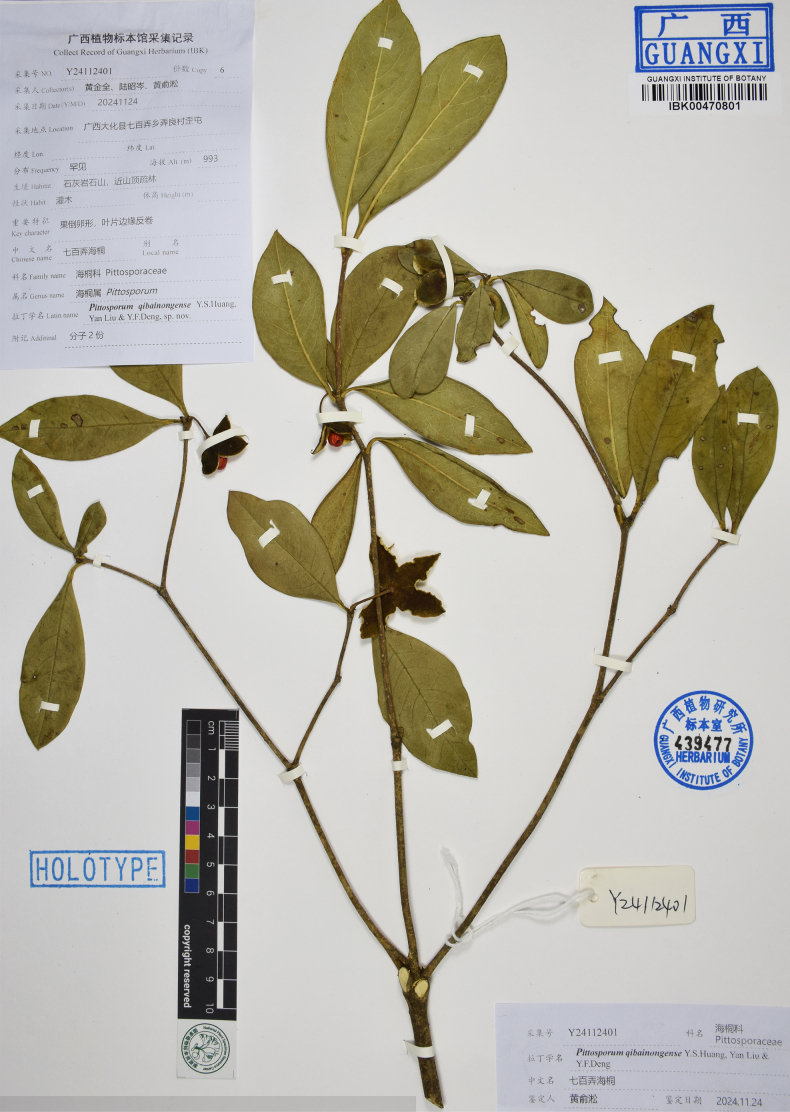
Holotype specimen of *Pittosporum qibainongense* (IBK).

#### Type.

China • Guangxi Zhuang Autonomous Region: Hechi City, Dahua County, Qibainong Town, Nongliang Village, Waixian, alt. 993 m, 24 November 2024, *J.Q.Huang, Z.C.Lu & Y.S.Huang Y24112401* (holotype: IBK00470801!, isotypes: IBK00470802!, IBSC!, PE!).

**Figure 2.**
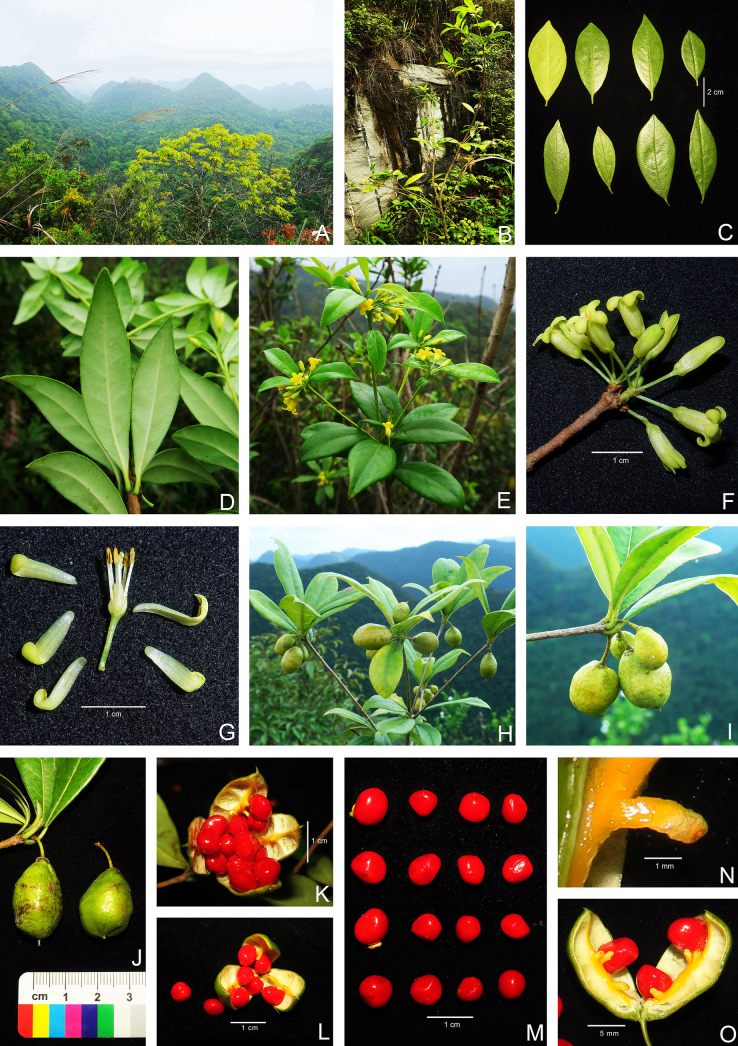
*Pittosporum qibainongense*. **A**. Habitat; **B**. Habit; **C, D**. Leaf blades (abaxial and adaxial view); **E**. Flowering branch; **F**. Flowering inflorescence; **G**. Floral anatomy (show pistil, stamens, calyces, and petals); **H, I**. Fruiting branch; **J**. Fruits; **K**. Mature fruit dehiscing by four valves and seeds; **L**. Mature fruit dehiscing by three valves and seeds; **M**. Seeds; **N**. Funicle; **O**. Opened fruit segments (show seeds and funicles).

#### Description.

***Evergreen shrubs***, 1.5–3 m tall. ***Branchlets*** sparsely puberulous when young, then glabrescent, sparsely lenticellate. ***Leaves*** alternate, clustered at branchlet apex; petiole 6–8 mm long, flat, the upper part narrow-winged, glabrous; blades leathery, obovate or oblong-lanceolate, 5–9 cm long, 1.5–4 cm wide, apex mucronate, acuminate or obtuse, margin entire, strongly revolute after drying, base cuneate, slightly decurrent, adaxially dark green, shiny, glabrous, abaxially pale green, sparsely puberulous when young, then glabrescent, midvein concave adaxially, prominent abaxially; lateral veins 6–9 pairs, slightly concave adaxially, prominent abaxially, curved arches meet near the margin, reticulate veins inconspicuous on both surfaces. ***Inflorescences*** borne on leaf axils at branchlet apex, umbellate, 2–8-flowered; pedicels 1–1.5 cm long, glabrous or sparsely puberulous; bracts ovate or ovate-lanceolate, 2–4 mm long, ca. 1.5 mm wide, margin ciliate; sepals 5, free, ovate-lanceolate, lanceolate, rarely ovate, 2–4 mm long, 1–2 mm wide, margin ciliate; petals 5, free, yellow or pale yellow, 1–1.5 cm long, 2–3 mm wide, apex reflexed, glabrous; stamens 5, 7–8 mm long, filaments glabrous, anthers dorsifixed, arrow-shaped; pistil 6–9 mm long, ovary densely puberulous, carpels 3, rarely 4, placentas at lower middle part, each carpel with 3–8 ovules, style 3–5 mm long, glabrous. ***Capsule*** ovate or obovoid, 1.5–3 cm long, 1–1.6 cm in diam., glabrous, rough when dry, dehiscing by 3 (or rarely 4) valves; pericarp 1.2–1.8 mm thick; seeds 10–25, red, nearly globose, or irregular polygon, 6–8 mm long, 5–7 mm in diam.; funicle flat, 2–3 mm long.

**Figure 3.**
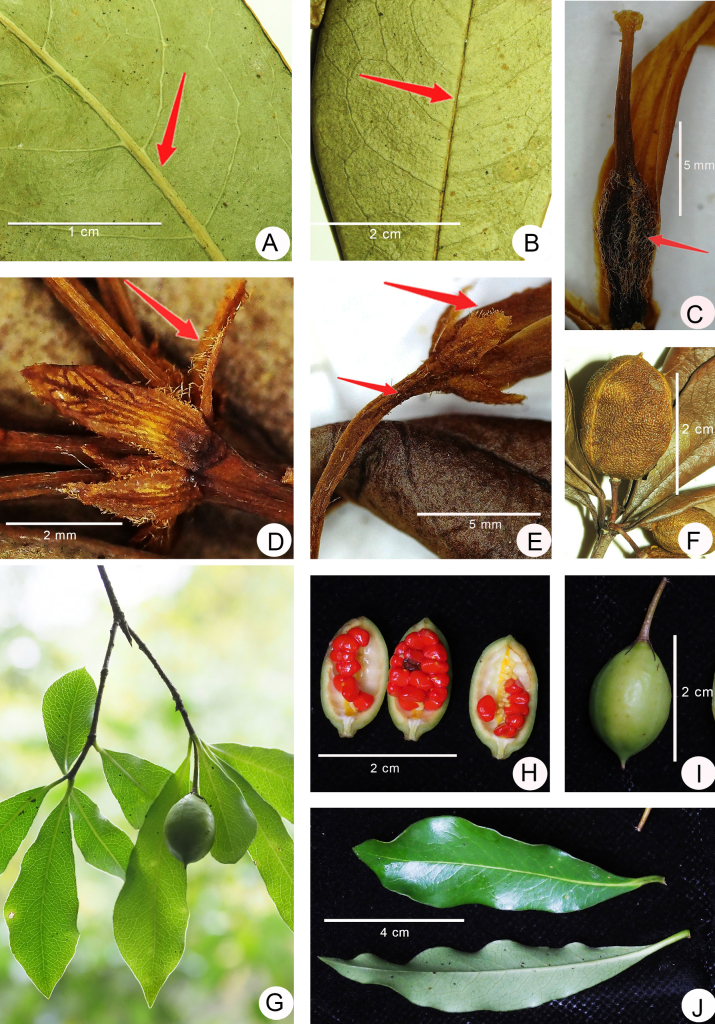
**A–F**. *Pittosporum qibainongense*. **A**. Abaxial leaf blade (showing midvein and lateral veins prominent); **B**. Adaxial leaf blade (showing midvein and lateral veins slightly concave); **C**. Pistil (show ovary densely puberulous and style glabrous); **D**. Bracts (show margin ciliate); **E**. Pedicel and sepals (show pedicel glabrous or sparsely puberulous, and sepals margin ciliate); **F**. Fruit (show the surface rough when dry); **G–J**. *P. trigonocarpum*. **G**. Fruiting branch; **H**. Mature fruit dehiscing by three valves and seeds; **I**. Fruit; **J**. Leaf blades (show abaxial and adaxial surfaces) (**G–J**. Photographed by Qin-Wen Lin).

#### Phenology.

*Pittosporum qibainongense* was observed flowering from April to May and fruiting from October to December.

#### Etymology.

The specific epithet refers to the type locality, Qibainong National Geopark in Guangxi, China. The Chinese name is given as “七百弄海桐 (pinyin: qī bǎi nòng hǎi tǒng).”

#### Distribution and habitat.

Thus far, *Pittosporum qibainongense* is only known from its type locality, Dahua County of Guangxi, China. It grows near the peak of limestone hills or hillside at an elevation of ca. 1000 m. The slope direction is to the southeast, and the slope gradient is ca. 50°. The tree layer is up to 8 m tall with a canopy cover of 85%, and the shrub and herb layer covers are 65% and 25%, respectively. The symbiotic plants include *Carpinus rupestris* A.Camus (Betulaceae), *Myrsine seguinii* H.Lév. (Primulaceae), *Platycarya strobilacea* Siebold & Zucc. (Juglandaceae), *Fraxinus insularis* Hemsl. (Oleaceae), *Pittosporum tonkinense* Gagnep. and *P. lenticellatum* Chun ex H.Peng & Y.F.Deng (Pittosporaceae), *Pistacia weinmanniifolia* J.Poiss. ex Franch. (Anacardiaceae), *Viburnum triplinerve* Hand.-Mazz. (Viburnaceae), *Alyxia sinensis* Champ. ex Benth. (Apocynaceae), *Leptodermis hechiensis* R.J.Wang (Rubiaceae), *Acer sycopseoides* Chun (Sapindaceae), *Sageretia thea* (Osbeck) M.C.Johnst. (Rhamnaceae), and *Miscanthus sinensis* Andersson (Poaceae).

#### Conservation status.

Although field surveys have been conducted in the karst areas of Dahua County for more than 10 years, no more than five subpopulations have been found at the type locality and its vicinity. It is obvious that its Extent of Occurrence (EOO) is relatively narrow, the distribution points are rare, and the Area of Occupancy (AOO) is extremely limited. Based on field surveys of the subpopulations, there are phenomena such as deforestation and grazing by local residents and environmental pollution caused by tourism development in their habitat. The influence of these factors is still ongoing. Therefore, we speculate that its habitat quality and population size have a downward trend. According to the Guidelines for Using the IUCN Red List Categories and Criteria (IUCN 2024), *P. qibainongense* is assessed as Endangered (EN) based on criteria [B1ab(iii, v)+2ab(iii, v)] (IUCN 2012).

#### Additional specimens examined

**(paratypes)**. China • Guangxi Zhuang Autonomous Region: Hechi City, Dahua County, Qibainong Town, Nongliang Village, Waixian, alt. 1050 m, 5 October 2023, *W.B.Xu, Z.C.Lu, M.L.Chang & J.Q.Huang 17576* (IBK); • the same locality, alt. 1000 m, 4 November 2023, *M.X.Li LMX0106* (IBK); • the same locality, alt. 1130 m, 11 April 2024, *W.B.Xu, Z.C.Lu, M.L.Mo, S.L.Chang & J.Q.Huang 18086* (IBK, IBSC); • the same locality, alt. 910 m, 11 April 2024, *W.B.Xu, Z.C.Lu, M.L.Mo, S.L.Chang & J.Q.Huang 18088* (IBK, PE); • the same locality, alt. 1020 m, 11 April 2024, *W.B.Xu, Z.C.Lu, M.L.Mo, S.L.Chang & J.Q.Huang 18057* (IBK).

#### Notes.

The morphological characteristics of the genus *Pittosporum* are highly heteroblastic due to differences in habitats or different growth periods, such as leaf size and shape, inflorescence and infructescence structure, etc. (Cayzer et al. 2023).

However, the number of parietal placentas is an important taxonomic characteristic of the genus *Pittosporum*. Morphologically, *P. qibainongense* is also similar to *P. podocarpum*, *P. omeiense*, and *P. kweichowense* in leaf shape, umbellate inflorescences, and the same number of parietal placentas, in addition to *P. trigonocarpum*. However, it can be easily distinguished from *P. podocarpum* by petioles 6–8 mm long (vs. 8–15 mm long), pedicels 1–1.5 cm long, glabrous or sparsely puberulous (vs. 2–3 cm long, glabrous), capsules glabrous, stipe inconspicuous (vs. puberulous, stipe 5–8 mm long), seeds 10–25 (vs. 8–10), funicle 2–3 mm long (vs. 3–4 mm long); from *P. omeiense* by lateral veins 6–9 pairs (vs. ca. 5 pairs), capsules ovate or obovoid (vs. ellipsoid), seeds 10–25, 6–8 mm long (vs. 7–8, 3–4 mm long); and from *P. kweichowense* by lateral veins 6–9 pairs, conspicuous on both surfaces (vs. 5–6 pairs, inconspicuous on both surfaces), petioles 6–8 mm long (vs. ca. 5 mm long), pedicels 1–1.5 cm long (vs. 4–7 mm long), ovary densely puberulous (vs. glabrous or sparsely puberulous), capsules multiple or solitary, 1.5–3 cm long, glabrous, stipe inconspicuous (vs. solitary, 1–1.5 cm long, puberulous, stipe less than 2 mm long), seeds 10–25 (vs. 6–8). A detailed morphological comparison among them is summarized in Table 1. A key to *P. qibainongense* and its morphologically similar species is provided below.

**Table 1.** A morphological comparison of key characteristics of *Pittosporum qibainongense* and its similar species.

Characters	*P. qibainongense*	*P. trigonocarpum*	*P. kweichowense*	*P. podocarpum*	*P. omeiense*
Leaf blade	5–9 × 1.5–4 cm; lateral veins 6–9 pairs, conspicuous on both surfaces	7–14 × 2.5–4 cm; lateral veins 6 pairs, inconspicuous on both surfaces	3–8 × 0.5–2.2 cm; lateral veins 5–6 pairs, inconspicuous on both surfaces	7–13 × 2–4 cm; lateral veins 6–8 pairs, conspicuous on both surfaces	7–10 × 1.5–2.5 cm; lateral veins ca. 5 pairs, conspicuous on both surfaces
Petiole	6–8 mm long	ca. 1 cm long	ca. 5 mm long	8–15 mm long	5–8 mm long
Pedicel	1–1.5 cm long, glabrous, or sparsely puberulous	1–2.5 cm long, glabrous	4–7 mm long, puberulous	2–3 cm long, glabrous	1.5–2 cm long, puberulous
Sepal	ovate-lanceola te or lanceolate, rarely ovate, 2–4 mm long	ovate, ca. 2 mm long	triangular-ovate, 1–1.5 mm long	ovate, ca. 3 mm long	long ovate, ca. 3 mm long
Ovary	densely puberulous	densely puberulous	densely brown puberulous	densely brown villous	glabrous, sparsely puberulous
Capsule	multiple or solitary, ovate or obovoid, 1.5–3 cm long, glabrous, stipe inconspicuous	solitary, ellipsoid, triangular, or globose, 2.5–2.7 cm long, puberulous, stipe less than 2 mm	solitary, obovoid or cylindric, 1–1.5 cm long, puberulous, stipe less than 2 mm	pear-shaped, ellipsoid, or long ellipsoid, 2–3 cm long, puberulous, stipe 5–8 mm long	ellipsoid, 1.6–2 cm long, glabrous, stipe inconspicuous
Funicle	at lower middle part of placenta	scattered on the elongated placenta	in middle part of placenta	in middle part of placenta	in middle part of placenta
Seed	10–25; 6–8 mm long	9–15; 5–6 mm in diam.	6–8; 5–6 mm in diam.	8–10; 6–7 mm long	7–8; 3–4 mm long
Funicle	2–3 mm long	ca. 2 mm long	2–3 mm long	3–4 mm long	ca. 2 mm long

The floral dimorphism of *Pittosporum* is an interesting phenomenon. Most species of *Pittosporum* have functionally unisexual flowers. However, the flowers of *P. qibainongense* are bisexual. In fact, all species of *Pittosporum* in China are bisexual, and only a tendency for separation of sexes has been observed in a few species. Previous studies have shown that species of *Pittosporum* are evolving from functional dioecism to monoecism (Schlessmann 2010; Schlessman et al. 2014). *P. qibainongense* is an evergreen shrub and can be used as an excellent landscaping tree because of its beautiful tree form, flowers, and fruits. It is adaptive to karst areas and has important application value in karst mountain greening.

##### Key to *P. qibainongense* and its similar species

**Table:** 

1a	Ovary puberulous, seeds more than 5 mm long.	
2a	Capsule pear-shaped, ellipsoid, or long ellipsoid, seeds 9–12, stipe 5–8 mm long	***P. podocarpum***
2b	Capsule ovate, obovoid, triangular, or cylindric, not pear-shaped, ellipsoid, or long ellipsoid, stipe inconspicuous or less than 2 mm	**3a**
3a	Capsule more than 1.5 cm long, petiole 6–10 mm long	**4a**
4a	Margin of leaf blade obviously revolute when dry, capsule multiple or solitary, ovate or obovoid, glabrous, funicle at lower middle part of placenta, seeds 10–25	***P. qibainongense***
4b	Margin of leaf blade flat when dry, capsule solitary, ellipsoid, triangular, or globose, pubescent, funicle scattered on the elongated placenta, seeds 9–15	***P. trigonocarpum***
3b	Capsule less than 1.5 cm long, petiole ca. 5 mm long	***P. kweichowense***
1b	Ovary glabrous, or sparsely puberulous, seeds 3–4 mm long	***P. omeiense***

## Supplementary Material

XML Treatment for Pittosporum qibainongense

## Additional information

### Use of AI

No use of AI was reported.

### Author ORCIDs

Yu-Song Huang https://orcid.org/0000-0003-2693-8518

Zhao-Cen Lu https://orcid.org/0000-0002-1725-0247

Yan Liu https://orcid.org/0000-0001-8931-9656

Yun-Fei Deng https://orcid.org/0000-0002-0876-3286

### Data availability

All of the data that support the findings of this study are available in the main text.
